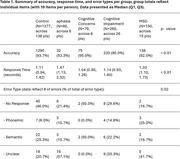# An Evaluation of the Remotely Administered MASTERMIND Assessment of Naming

**DOI:** 10.1002/alz70856_100512

**Published:** 2025-12-25

**Authors:** Rene L Utianski, Joseph R Duffy, Leland Barnard, John L. Stricker, Ronald Petersen, David T. Jones, Hugo Botha

**Affiliations:** ^1^ Mayo Clinic, Rochester, MN, USA; ^2^ Department of Neurology, Mayo Clinic, Rochester, MN, USA; ^3^ Department of Information Technology, Mayo Clinic, Rochester, MN, USA

## Abstract

**Background:**

To promote equitable access to neurologic diagnostics, our team developed MASTERMIND—the Mobile Assessment of Speech To Evaluate, Recognize, and Monitor Indicators of Neurologic health and Disease. One subtest, the MASTERMIND Assessment of Naming, includes ten pictures randomly selected from a list of 45 words varying in frequency. This study aimed to 1) evaluate the feasibility of a remotely self‐administered naming test for patients with cognitive, linguistic, and/or communication difficulties and 2) assess the sensitivity and specificity of performance measures (accuracy, error type, and response time) for detecting aphasia and cognitive‐communication difficulties.

**Method:**

The task was remotely administered to 138 controls, 26 cognitively impaired individuals, 15 with motor speech disorders, 8 with cognitive concerns, and 6 with aphasia. Participants named pictures displayed on‐screen, advancing through the task by clicking “Next.” Responses were processed using natural language processing (NLP). Audio was transcribed with Whisper Large‐v3, and responses were matched to stimuli within a 0.4‐second threshold. Errors were categorized by type (semantic, phonemic, or unclear source) through consensus between two raters. Performance metrics (accuracy, error type, response time) were analyzed using Kruskal‐Wallis and Pearson's Chi‐Square tests, with AUROC analysis evaluating diagnostic performance.

**Results:**

Differences in accuracy, response time, and error type were observed across groups (Table 1). Controls and cognitively impaired participants performed similarly, with longer response times observed in patients with aphasia and motor speech disorders. Accuracy alone was a poor predictor of all patients versus controls (AUROC = 0.468) but prediction was better in distinguishing aphasia patients from 1) controls (AUROC = 0.698), 2) cognitively impaired participants (AUROC = 0.679), and 3) those with MSDs (AUROC = 0.670).

**Conclusions:**

The MASTERMIND Assessment of Naming is a feasible, scalable, self‐administered diagnostic tool enhanced by automated NLP. It provides critical insights through response time, accuracy, and error analysis. Limitations include potential bias in error classification and fixed processing thresholds. The study highlights MASTERMIND's potential for patient triaging and screening. Future research will evaluate the sensitivity of combined performance metrics to disease progression, expand sample sizes and diversity, and refine usability based on participant feedback.